# QTL mapping: insights into genomic regions governing component traits of yield under combined heat and drought stress in wheat

**DOI:** 10.3389/fgene.2023.1282240

**Published:** 2024-01-10

**Authors:** Karthik Kumar Manjunath, Hari Krishna, Narayana Bhat Devate, V. P. Sunilkumar, Sahana Police Patil, Divya Chauhan, Shweta Singh, Sudhir Kumar, Neelu Jain, Gyanendra Pratap Singh, Pradeep Kumar Singh

**Affiliations:** ^1^ Division of Genetics, ICAR-Indian Agricultural Research Institute, New Delhi, India; ^2^ Division of Plant Physiology, ICAR-Indian Agricultural Research Institute, New Delhi, India; ^3^ National Bureau of Plant Genetic Resources, New Delhi, India

**Keywords:** wheat, quantitative trait locus, heat, drought, combined stress

## Abstract

Drought and heat frequently co-occur during crop growth leading to devastating yield loss. The knowledge of the genetic loci governing component traits of yield under combined drought and heat stress is essential for enhancing the climate resilience. The present study employed a mapping population of 180 recombinant inbred lines (RILs) derived from a cross between GW322 and KAUZ to identify quantitative trait loci (QTLs) governing the component traits of yield under heat and combined stress conditions. Phenotypic evaluation was conducted across two consecutive crop seasons (2021–2022 and 2022–2023) under late sown irrigation (LSIR) and late sown restricted irrigation (LSRI) conditions at the Indian Council of Agricultural Research Institute–Indian Agricultural Research Institute (ICAR-IARI), New Delhi. Various physiological and agronomic traits of importance were measured. Genotyping was carried out with 35K SNP Axiom breeder’s genotyping array. The linkage map spanned a length of 6769.45 cM, ranging from 2.28 cM/marker in 1A to 14.21 cM/marker in 5D. A total of 35 QTLs were identified across 14 chromosomes with 6B containing the highest (seven) number of QTLs. Out of 35 QTLs, 16 were major QTLs explaining the phenotypic variance greater than 10%. The study identified eight stable QTLs along with two hotspots on chromosomes 6B and 5B. Five QTLs associated with traits thousand-grain weight (TGW), normalized difference vegetation index (NDVI), and plant height (PH) were successfully validated. Candidate genes encoding antioxidant enzymes, transcription factors, and growth-related proteins were identified in the QTL regions. *In silico* expression analysis highlighted higher expression of transcripts TraesCS2D02G021000.1, TraesCS2D02G031000, TraesCS6A02G247900, and TraesCS6B02G421700 under stress conditions. These findings contribute to a deeper understanding of the genetic architecture underlying combined heat and drought tolerance in wheat, providing valuable insights for wheat improvement strategies under changing climatic conditions.

## Introduction

Over the past few years, agricultural production in tropical and subtropical areas has encountered substantial difficulties attributed to the increasing effects of global warming and climate change. These changes have given rise to an array of new biotic and abiotic stresses emerging as prominent concerns. Among these stresses, combined heat and drought stress has proven particularly severe and causes detrimental effects on crop growth and productivity (Abbass et al., 2022).

In the Indian scenario, most of the wheat-growing regions that are located in the central and peninsular areas consistently experience heat stress throughout the crop season. Conversely, the north western and north eastern plain zones experience terminal heat stress due to delayed sowing practices. Approximately 13.5 million hectares of wheat cultivation areas are affected by heat stress (Pandey et al., 2021). The optimal temperature for grain filling and development during the post-anthesis phase is 22°C–25°C. Deviations from this range, particularly higher temperatures, result in irreversible heat-induced damage. Evidently, every 1°C increase above this threshold cuts grain filling duration by 2.8 days (Streck, 2005), reduces grain numbers by 4% (Fischer, 1985), decreases grain weight by 5%, and ultimately leads to a reduction in production up to 18% (Dubey et al., 2020). Elevated temperature and drought stress often coincide with the critical grain-filling period of wheat growth. In dryland and rain-fed zones, the simultaneous occurrence of high temperature and low moisture significantly shortens the grain-filling period, resulting in forced maturity (Hlaváčová et al., 2018). The combined stress exerts intricate effects through multifaceted mechanisms resulting in physiological changes and biochemical alterations (Fahad et al., 2017). In these challenging scenarios, the primary objective is to identify genotypes capable of withstanding concurrent heat stress and water deficit conditions (Reynolds et al., 2012).

Improving plants' ability to withstand heat and drought through traditional breeding methods is challenging. These challenges arise due to the complex nature of inheritance of the traits. Furthermore, accurate phenotyping of these traits is difficult as they are influenced by spatial and temporal variations. The modest heritability coupled with the unpredictable nature of yield diminishes the efficacy of conventional breeding approaches (Bhusal et al., 2017; Devate et al., 2023). The understanding of the various physiological and biochemical mechanisms controlling stress responses is crucial for identifying specific traits that confer drought and heat adaptation. However, assessing biochemical traits in a large number of germplasms is a challenging and laborious task. The agronomic traits such as plant height, spike length, number of spikelets, grain weight per spike, and thousand-grain weight can be measured relatively easily, and hence these traits can be targeted for developing more stress-tolerant crop varieties (Devate et al., 2022). The identification of quantitative trait loci (QTLs) associated with intricate traits furnishes invaluable insights into the chromosomal regions regulating these characteristics (Devate et al., 2022; Manjunath et al., 2023). Genomics tools enable the identification of potential candidate genes within these regions (Shavrukov, 2016). Molecular markers linked to component traits of stress tolerance help in the integration of genomic regions into elite varieties to enhance climate resilience (Sunilkumar et al., 2023). However, the challenge lies in identifying stable marker–QTL associations across diverse genetic backgrounds and environments given the heterogeneity of growth conditions (Mourad et al., 2018). Confirming the validity of these QTLs in the diverse germplasms is essential for establishing their practical utility through marker-assisted selection (Gautam et al., 2015; Todkar et al., 2020).

While advancements in wheat research have predominantly focused on identifying QTLs under individual stresses such as high temperature or drought, the co-occurrence of these stresses in natural conditions has been relatively overlooked. Consequently, limited investigations have been conducted to identify and validate QTLs governing component traits of yield under combined heat and drought stress conditions (Tahmasebi et al., 2016; Schmidt et al., 2020; Khaled et al., 2022). In the current study, we aim to identify and validate QTLs governing component traits of yield under heat and combined stress in wheat. The validated QTLs hold great promise for practical plant breeding efforts ultimately leading to the development of superior wheat lines with enhanced resistance to combined stress conditions.

## Materials and methods

### Plant materials

The mapping population comprises 180 recombinant inbred lines (RILs) developed from a cross between GW322 and KAUZ. KAUZ is a derivative of a synthetic wheat variety developed by CIMMYT and known for abiotic stress tolerance. The variety GW322 is released for cultivation under timely sown irrigated condition (TSIR) in the central zone of India which has poor performance under high-temperature and moisture deficit stress. The validation population consisting of 166 RILs derived from the cross between HD3086 and HI1500 was used. HD3086 is a hexaploid wheat variety developed by the Indian Agricultural Research Institute (IARI), New Delhi, India, for cultivation under timely sown and irrigated conditions but performs poorly under drought and heat stress (Singh et al., 2014). By contrast, HI1500 is a widely accepted wheat variety recommended for cultivation under restricted irrigation conditions in the parts of the central zone in India. This variety possesses valuable attributes associated with resistance to drought and heat stress (Sunilkumar et al., 2022).

### Treatment details and phenotyping

The experiment was carried out at the Indian Council of Agricultural Research Institute–Indian Agricultural Research Institute (ICAR-IARI), New Delhi (28.6550°N, 77.1888°E, MSL 228.61 m), India, over two consecutive crop seasons (2021–2022 and 2022–2023). The study involved the evaluation of RILs developed from GW322/KAUZ along with their parents under two distinct conditions: heat stress (late sown irrigation, LSIR) and combined drought and heat stress (late sown restricted irrigation, LSRI) in augmented design (Federer, 1956; Federer, 1961; Searle, 1965). The experimental field was divided into four blocks, with four checks utilized in the study. Each check was replicated thrice. Additionally, the validation population was evaluated in alpha-lattice design with two replications under timely sown restricted irrigation (TSRI), TSIR, LSIR, and LSRI conditions. The details of the treatment conditions are given in Table 1. Each genotype was sown in a plot of size 0.68 m^2^. Each genotype was sown in three rows, each of which was 1 m in length. Uniform agronomic practices were followed for the establishment, except for the specific treatments under investigation. The weather parameters during crop season are given in Supplementary Table S1. The component traits of yield such as days to heading (DH), normalized difference vegetation index (NDVI), SPAD chlorophyll content (SPAD), plant height (PH), spike length (SL), thousand-grain weight (TGW), grain weight per spike (GWPS), biomass (BM), and grain yield per plot (PY) were measured under heat and combined stress conditions. The SPAD meter functions by emitting a specific wavelength of light, usually in the red and infrared light spectra. It measures the amount of light absorbed by chlorophyll in the leaf and calculates a numerical SPAD reading, which reflects the chlorophyll concentration. SPAD values were recorded at the anthesis stage. The instrument GreenSeeker™ was used to record the NDVI which measures by analyzing the difference between the reflectance of near-infrared (NIR) and red light (Red). The NDVI was measured at four stages: at anthesis, 10 days after anthesis, 20 days after anthesis, and 30 days after anthesis.

$$\text{NDVI}=\left(\text{NIR}‐\text{Red}\right)/\left(\text{NIR}+\text{Red}\right).$$

**TABLE 1:** Details of treatment conditions imposed for evaluation of mapping and validation populations.

Location	Condition		Treatment
Delhi	Timely sown (first fortnight of November)	Irrigated (six irrigations)	Control (TSIR)
		Restricted irrigation (two irrigations)	Drought (TSRI)
	Late sown (second fortnight of December)	Irrigated (six irrigations)	Heat (LSIR)
		Restricted irrigation (two irrigation)	Combined stress (LSRI)

### Phenotypic data analysis

The analysis of variance (ANOVA) was conducted using the PBTools v1.4 software (PBTools, version 1.4, 2014) and MetaRv6.0 (Multi-Environment Trial Analysis with R) software (Alvarado et al., 2020). Descriptive statistics that includes mean, range, coefficient of variation, and least significant difference was calculated. Genetic parameters such as genotypic variance and heritability were estimated. The best linear unbiased predictors (BLUPs) (Robinson, 1991) were calculated for individual seasons and pooled over seasons using the MetaRv6.0 and PBTools v1.4 software. Correlation analyses were performed using RStudio v4.3.1 by using the “corr” package (RStudio Team, 2020).

### Genotyping and linkage map construction

**FIGURE 1:**
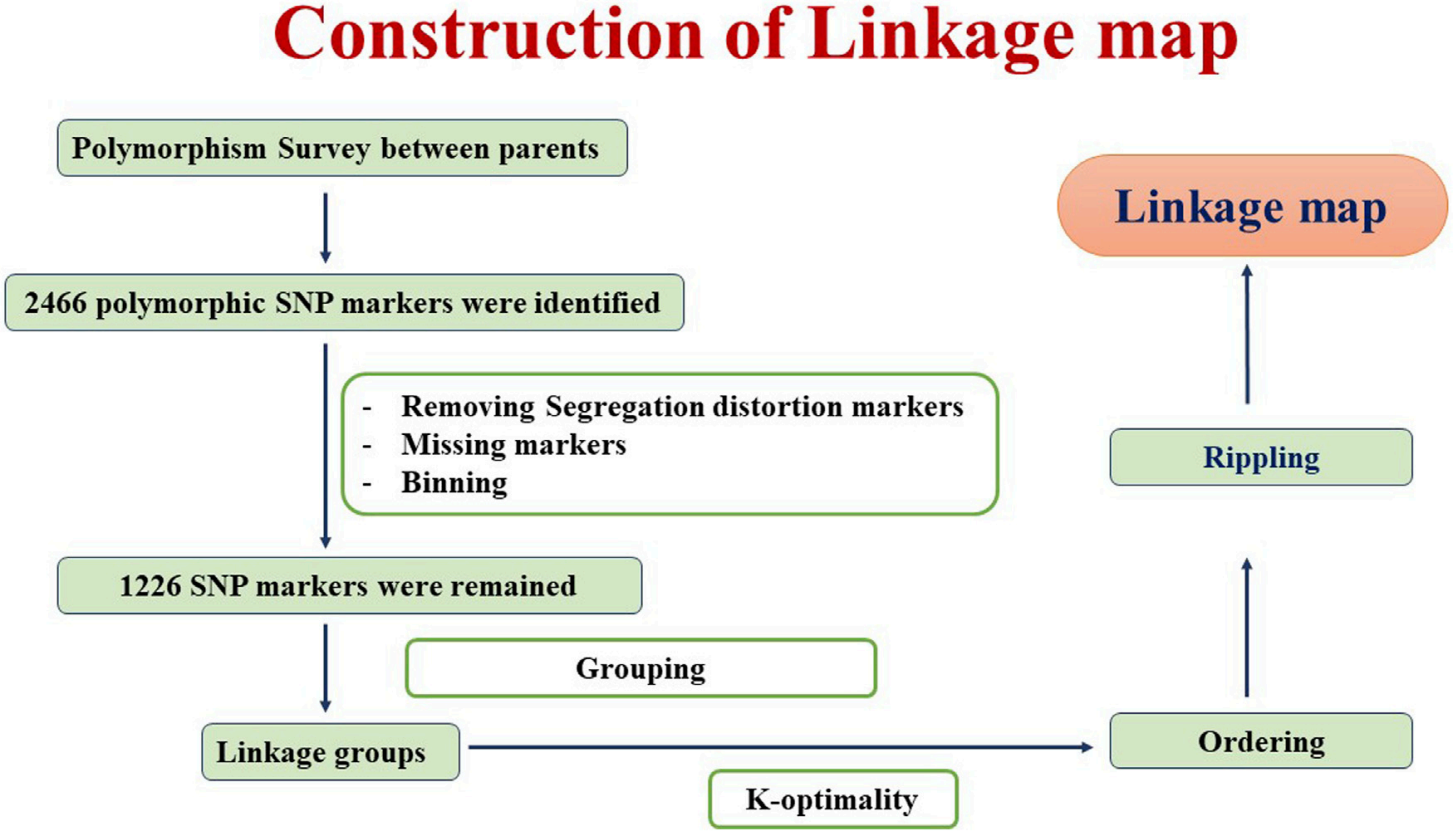
Procedure followed for linkage map construction using the QTL IciMapping software.

DNA was isolated from 21-day-old seedlings using the CTAB method (Murray and Thompson, 1980). DNA quality check was done using 0.8% agarose gel electrophoresis. The Axiom breeder’s array containing 35K single-nucleotide polymorphism (SNP) was employed for the genotyping of RILs and parents. The parental genotypic data were preprocessed to eliminate monomorphic markers, heterozygous markers, and markers with unknown positions. A total of 2,466 polymorphic markers between GW322 and KAUZ were identified. Among these, a total of 1,226 markers were retained for linkage map construction after the removal of markers that deviated from the Mendelian segregation ratio and binning. A linkage map was constructed using QTL IciMapping v4.2 (Meng et al., 2015), employing the Kosambi mapping function with a recombination fraction of 0.37 as the linkage criterion (Kosambi, 1944). Any two markers with an estimated recombination frequency lower than the threshold (0.37) had been grouped. The optimization algorithm K-optimality was used to determine marker order and map distances. Recombination-based rippling with a window size of 5 cM was used to improve the accuracy of the genetic map. The detailed flowchart of the linkage map construction is given in Figure 1.

### QTL analysis

QTL mapping was done using the IciMapping 4.2 software (Meng et al., 2015), adopting the inclusive composite interval mapping (ICIM-ADD) model (Wang, 2009). BLUP values and linkage map details were used for QTL mapping. Stepwise regression with a walking speed of 1.0 cM and a significant threshold of *p* = 0.001 was employed to search the QTLs. The threshold logarithm of odds (LOD) score was chosen by 1,000 permutations with 5% type 1 error to establish marker–trait linkage. QTL naming was done using the standard nomenclature (McIntosh et al., 2013), denoting them as “Q,” followed by trait abbreviation, research department name, and chromosome number.

### Putative candidate genes in QTL regions

The candidate genes (CGs) present within the genomic regions of the QTLs were listed using the BioMart tool in the Ensembl Plants website (https://plants.ensembl.org/index.html). Subsequently, a subset of CGs was predicted from the list based on their functional relevance to the trait. An *in silico* expression analysis of the predicted candidate genes was done using the “wheat expression database” (http://www.wheat-expression.com/) to investigate the expression of candidate genes.

### QTL validation

The RILs comprising 166 lines were used for validation. The genotyping of the validation population was done using SNP markers (35K Axiom breeder’s array). A single marker analysis was conducted to validate the markers. Based on the genotypic data of the linked marker allele, the validation population was subdivided into two groups ensuring each subgroup comprised individuals with the same allele. The significant difference between the means of the two allele groups was evaluated using single factor ANOVA to validate the marker effect on the trait. A low *p*-value (*p* < 0.05) indicated significant difference between the two groups, supporting the presence of SNP effects on the trait.

## Results

The phenotypic evaluation of the RILs was conducted under heat stress and combined stress. The crop was sown during the second fortnight of December, and the anthesis coincided with the high temperature during February–March. The rainfall during the crop period was 194.3 and 187.2, respectively, in 2021–2022 and 2022–2023 (Supplementary File S1). The genotypes showed significant reduction in NDVI, SPAD, PH, SL, BM, DH, GWPS, TGW, PY, and SN under combined stress conditions as compared to heat stress alone. Notably, the mean PH was significantly reduced under combined stress (84.90 cm) as compared to heat stress (98.45 cm). Similarly, BM and PY showed significantly lower mean values in combined stress (0.64 kg and 0.15 kg, respectively) than in heat stress (1.13 kg and 0.19 kg, respectively). These trends were consistent across the seasons (Supplementary File S2).

### ANOVA, heritability, and correlation analysis

**FIGURE 2:**
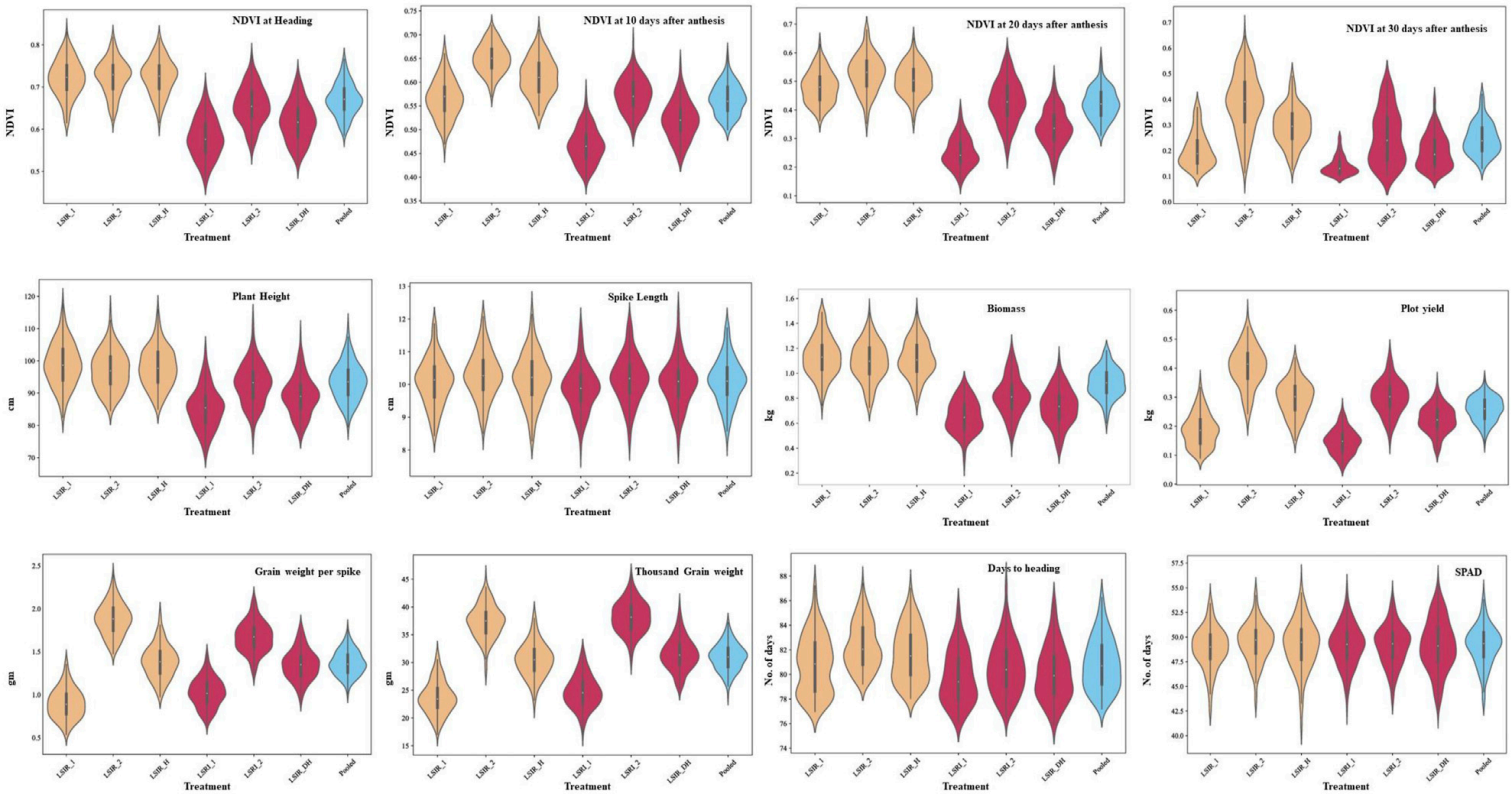
Frequency distribution for component traits of drought and heat tolerance in the RIL population under late sown irrigated and late sown restricted irrigated conditions.

**FIGURE 3:**
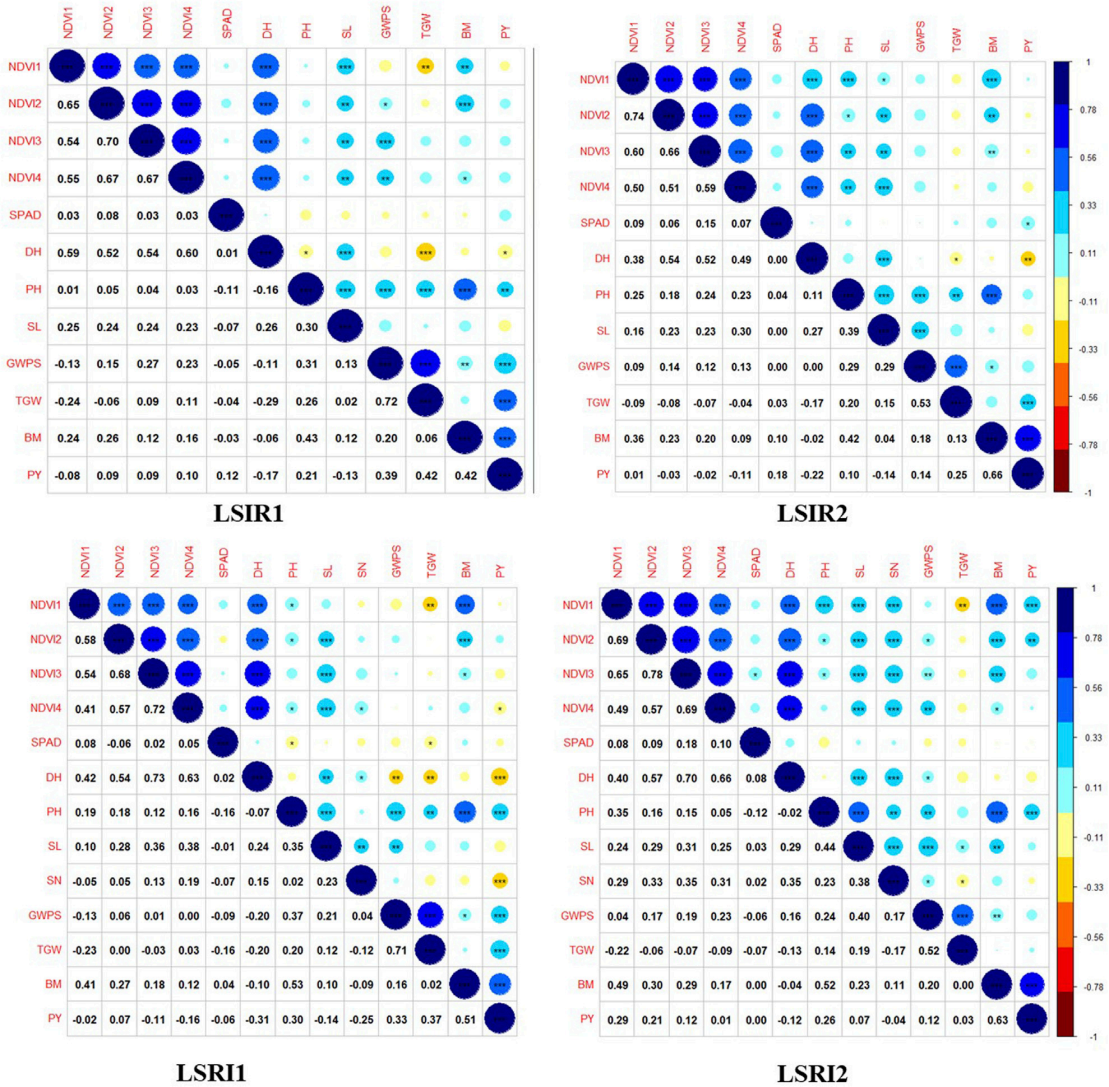
Correlation plots depicting associations among component traits yield under drought and heat tolerance.

The ANOVA revealed the presence of significant differences between the genotypes for all the traits studied. The majority of the traits exhibited a normal distribution as depicted in the violin plots, except NDVI at grain-filling stages (Figure 2). Heritability estimates indicated that traits, *viz.*, PH (84.54%), SL (73.30%), DH (82.96%), GWPS (68.81%), and TGW (70.04%), showed high heritability, while BM (26.52%) and PY (27.03%) showed low heritability (Supplementary File S2). Furthermore, a correlation analysis was conducted to explore the associations between traits. Under heat stress, traits PH (0.30***), GWPS (0.33***), TGW (0.37***), and BM (0.51***) showed significant positive correlations with PY, while DH (−0.31***) and SN (−0.25***) exhibited significant negative correlations. Similar associations among the traits were also observed in other treatment conditions and across years (Figure 3).

### Genetic map and QTL mapping

**FIGURE 4:**
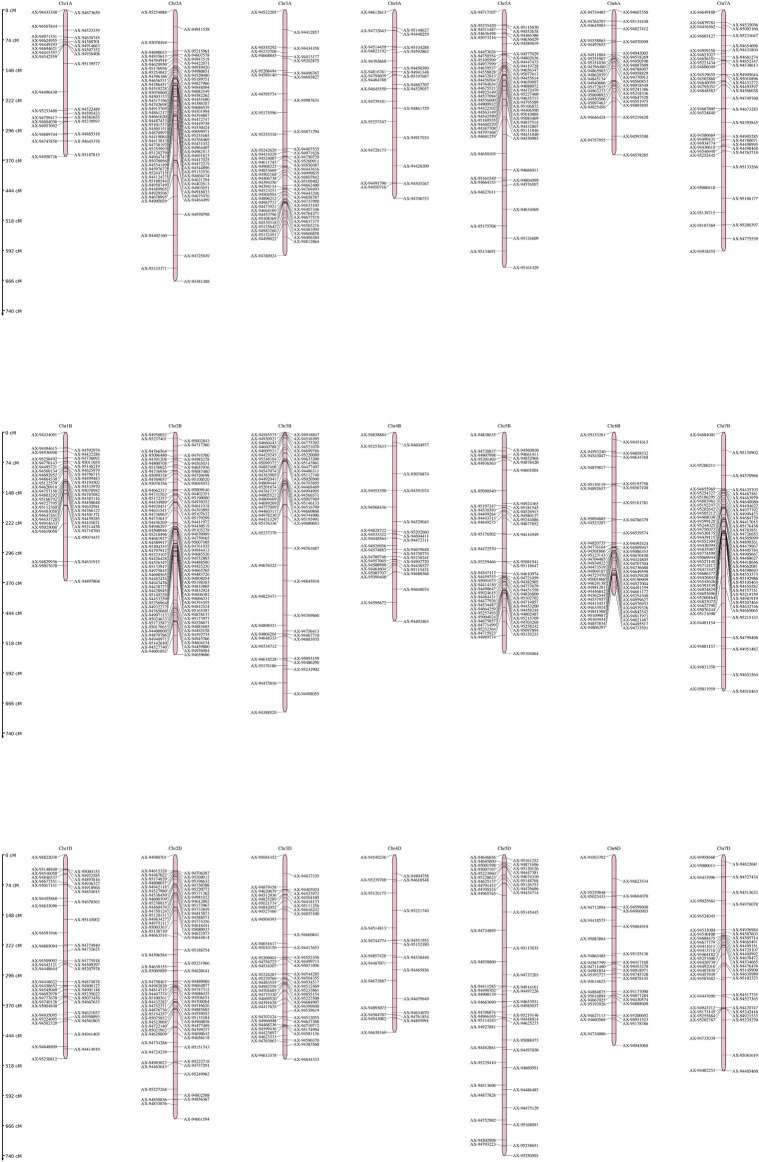
Genetic linkage map genome of the wheat genome developed using SNP markers in the RIL population of GW322/KAUZ.

The linkage map was constructed using SNP markers. A total of 2,466 polymorphic markers were identified between GW322 and KAUZ, out of which, 1,226 non-redundant markers uniformly distributed across all the 21 chromosomes were used for linkage map construction (Figure 4). The linkage map spanned a total length of 6,769.45 cM. The marker density ranged from 2.28 cM/marker in 1A to 14.21 cM/marker in 5D chromosome with an average of 5.52 cM/marker. The B genome carried the highest number of markers (514), followed by A (412) and D (300). Chromosome 2B had the highest number of markers, i.e., 124, while chromosome 1B had only 13 markers. The details of the linkage map are given in Table 2.

**TABLE 2:** Distribution of markers and map density across chromosomes in the linkage map developed in RILs of GW322/KAUZ.

Chromosome	Number of SNPs	Map distance (cM)	Map density (cM/marker)
1A	45	102.76	2.28
2A	48	295.88	6.16
3A	92	322.23	3.50
4A	35	300.11	8.57
5A	60	473.50	7.89
6A	54	375.63	6.96
7A	78	333.89	4.28
1B	13	174.37	13.41
2B	124	398.15	3.21
3B	117	285.89	2.44
4B	61	181.63	2.98
5B	66	368.55	5.58
6B	49	314.77	6.42
7B	84	307.90	3.67
1D	24	181.18	7.55
2D	84	407.48	4.85
3D	52	446.28	8.58
4D	28	358.72	12.81
5D	34	483.24	14.21
6D	29	384.24	13.25
7D	49	273.05	5.57
A Genome	412	2204.00	5.35
B Genome	514	2031.26	3.95
D Genome	300	2534.19	8.45
Total	1226	769.45	5.52

**FIGURE 5:**
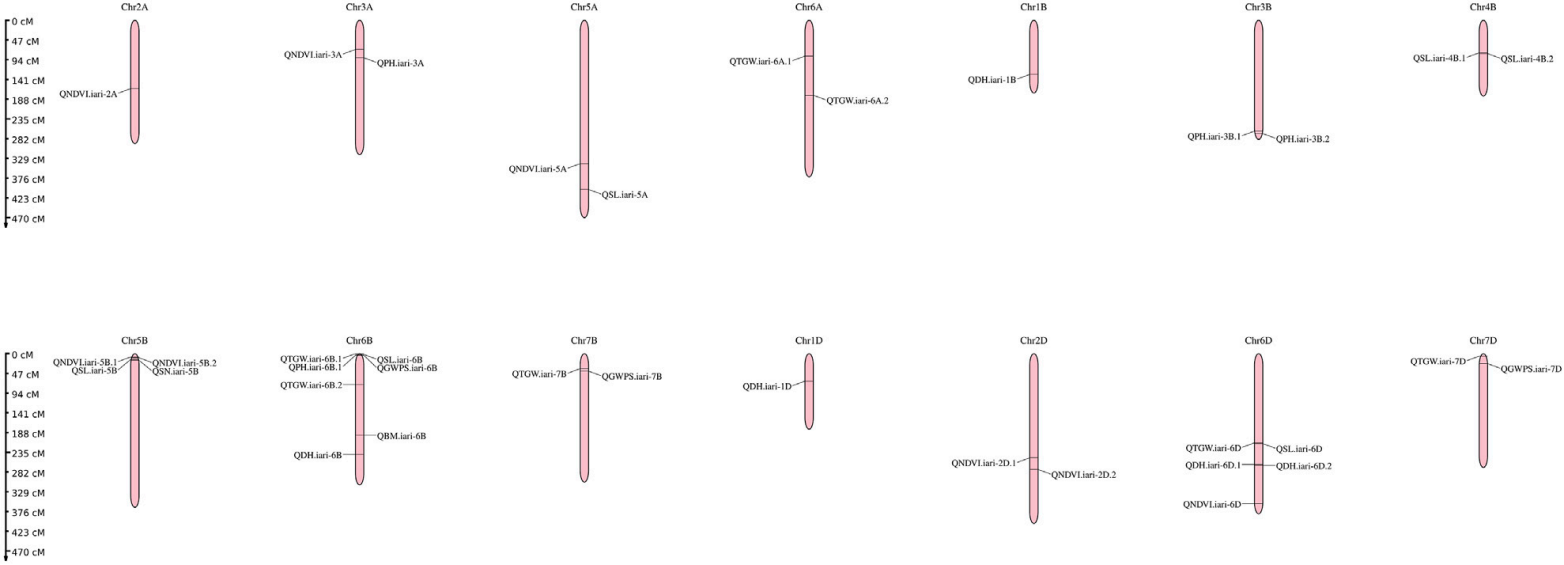
Genetic linkage map of the wheat genome depicting QTLs identified under late sown irrigated and late sown restricted irrigated conditions.

**FIGURE 6:**
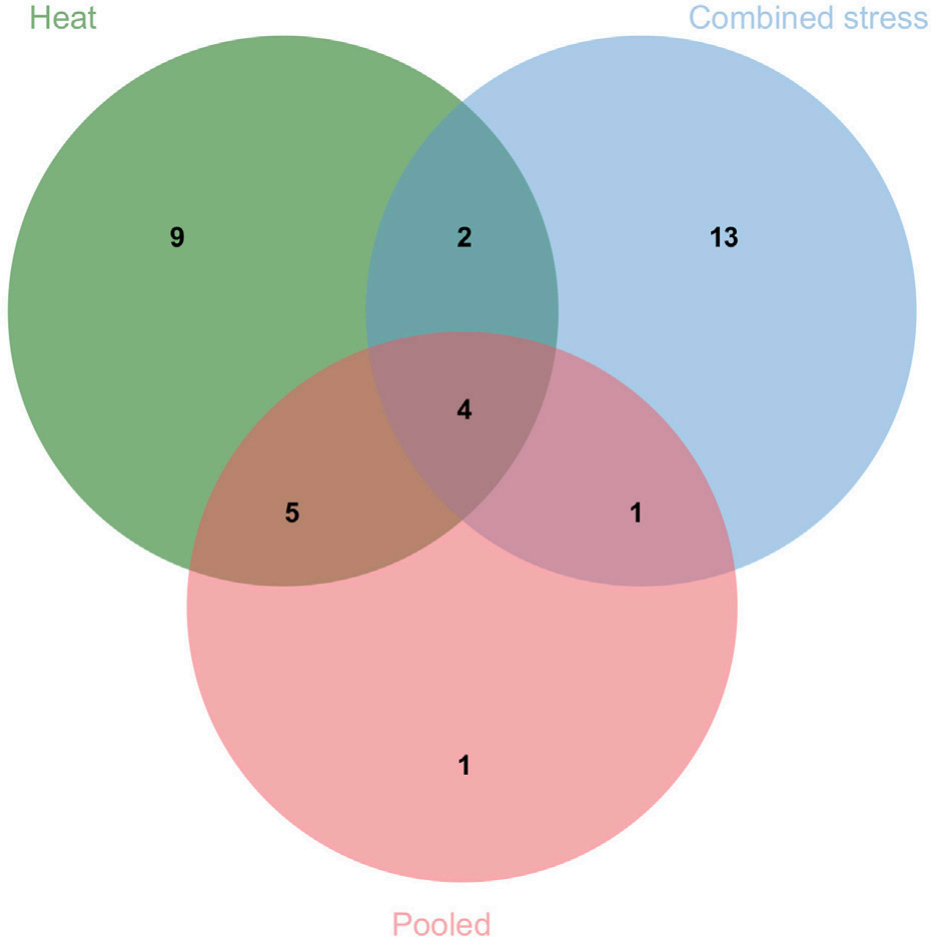
Venn diagram depicting the number of QTLs identified under individual and multiple treatments.

The present study identified 35 QTLs governing the component traits of yield under heat and combined stress. These QTLs were located on 14 chromosomes (Figure 5). Among the 35 QTLs, nine were identified under heat stress, 13 under combined stress, and one in the pooled mean condition. Several QTLs were detected across multiple conditions. Two QTLs were found in both heat stress and combined stress, one QTL appeared in combined stress and the pooled mean, five QTLs were identified in heat stress and the pooled mean conditions, and four QTLs consistently appeared in all conditions (Figure 6). Chromosome 6B displayed the highest number of QTLs (seven), followed by chromosome 6D (five), and chromosome 5B (four). In addition, chromosomes 3A, 5A, 6A, 3B, 4B, 7B, 2D, and 7D each carried two QTLs, while the remaining chromosomes 2A, 1B, and 1D carried a single QTL each. The list of QTLs identified for component traits of yield under heat and combined stress is given in Table 3.

**TABLE 3:** List of QTLs identified for component traits of heat and drought tolerance in RILs derived from GW322/KAUZ.

No.	QTL	Treatment	Year	Chr	Pos	Left marker	Right marker	LOD	PVE (%)	Add	Left CI	Right CI
Plant height												
1	*QPH.iari-3A*	Heat	2021–2022	3A	90	AX-94443246	AX-94578679	7.48	12.25	2.64	89.50	90.50
2	*QPH.iari-3B.1*	Heat	2022–2023	3B	265	AX-94498500	AX-94441567	10.46	19.70	3.62	257.50	271.50
		Pooled	Pooled	3B	266	AX-94498500	AX-94441567	8.74	15.83	2.86	255.50	271.50
		Heat	Pooled	3B	267	AX-94498500	AX-94441567	9.97	17.89	3.26	258.50	271.50
3	*QPH.iari-3B.2*	Drought and heat	2021–2022	3B	272	AX-94533181	AX-94509316	6.30	10.00	2.28	271.50	272.50
		Drought and heat	2022–2023	3B	272	AX-94533181	AX-94509316	4.33	8.69	2.03	271.50	277.50
		Drought and heat	Pooled	3B	272	AX-94533181	AX-94509316	5.92	9.54	2.10	271.50	278.50
4	*QPH.iari-6B.1*	Heat	2021–2022	6B	0	AX-94548199	AX-94998924	6.03	9.47	2.34	0.00	1.50
		Drought and heat	2021–2022	6B	0	AX-94548199	AX-94998924	7.19	11.10	2.42	0.00	1.50
		Drought and heat	2022–2023	6B	0	AX-94548199	AX-94998924	3.36	6.37	1.75	0.00	1.50
		Pooled	Pooled	6B	0	AX-94548199	AX-94998924	7.75	9.31	2.21	0.00	1.50
		Drought and heat	Pooled	6B	0	AX-94548199	AX-94998924	6.52	10.14	2.17	0.00	1.50
		Heat	Pooled	6B	0	AX-94548199	AX-94998924	7.28	8.84	2.30	0.00	1.50
		Heat	2022–2023	6B	1	AX-94548199	AX-94998924	5.79	6.13	2.03	0.00	1.50
Days to heading												
1	*QDH.iari-1B*	Heat	2021–2022	1B	129	AX-94897804	AX-94908088	6.35	5.14	−0.91	116.50	143.50
		Drought and heat	2021–2022	1B	129	AX-94897804	AX-94908088	7.62	6.37	−0.88	118.50	142.50
		Pooled	Pooled	1B	129	AX-94897804	AX-94908088	7.29	6.10	−0.84	117.50	144.50
2	*QDH.iari-6B*	Drought and heat	2022–2023	6B	241	AX-95109981	AX-94420407	5.84	6.00	0.97	234.50	250.50
3	*QDH.iari-1D*	Heat	2021–2022	1D	65	AX-94957467	AX-94517412	11.69	9.99	1.27	60.50	66.50
		Drought and heat	2021–2022	1D	66	AX-94957467	AX-94517412	11.48	9.81	1.08	62.50	66.50
		Pooled	Pooled	1D	66	AX-94957467	AX-94517412	11.13	9.54	1.04	61.50	66.50
4	*QDH.iari-6D.1*	Drought and heat	2022–2023	6D	266	AX-94977405	AX-95120610	12.80	10.57	−1.28	257.50	275.50
5	*QDH.iari-6D.2*	Drought and heat	Pooled	6D	268	AX-95120610	AX-94881172	4.57	10.35	−0.71	251.50	279.50
Grain weight per spike												
1	*QGWPS.iari-6B*	Heat	Pooled	6B	1	AX-94548199	AX-94998924	3.28	8.06	0.05	0.00	1.50
2	*QGWPS.iari-7B*	Drought and heat	2022–2023	7B	41	AX-94913939	AX-94843008	4.56	7.75	−0.06	40.50	41.50
		Pooled	Pooled	7B	41	AX-94913939	AX-94843008	3.57	7.29	−0.04	40.50	41.50
3	*QGWPS.iari-7D*	Heat	2021–2022	7D	23	AX-95217260	AX-94919750	4.11	8.67	−0.06	17.50	35.50
Thousand-grain weight												
1	*QTGW.iari-6A.1*	Pooled	Pooled	6A	86	AX-94825456	AX-94953201	5.53	11.16	−0.92	74.50	96.50
2	*QTGW.iari-6A.2*	Heat	2022–2023	6A	179	AX-94971148	AX-94964037	5.06	10.67	0.97	177.50	179.50
		Heat	Pooled	6A	179	AX-94971148	AX-94964037	6.17	12.57	1.12	178.50	179.50
3	*QTGW.iari-6B.1*	Heat	2021–2022	6B	0	AX-94548199	AX-94998924	5.01	10.11	1.00	0.00	1.50
		Drought and heat	2022–2023	6B	0	AX-94548199	AX-94998924	6.05	10.43	1.01	0.00	1.50
4	*QTGW.iari-6B.2*	Drought and heat	Pooled	6B	73	AX-94507146	AX-95103934	3.34	5.89	−1.23	59.50	86.50
5	*QTGW.iari-7B*	Drought and heat	2022–23	7B	35	AX-95652788	AX-94878591	5.16	9.23	−0.94	33.50	35.50
6	*QTGW.iari-6D*	2022–2023		6D	213	AX-95159098	AX-95023286	5.79	9.66	0.86	206.50	215.50
7	*QTGW.iari-7D*	Heat	2022–2023	7D	5	AX-94405992	AX-94640050	6.52	13.84	−1.10	3.50	5.50
Spike length												
1	*QSL.iari-5A*	Drought and heat	2021–2022	5A	406	AX-95259552	AX-94730816	3.45	8.66	−0.24	393.50	411.50
2	*QSL.iari-4B.1*	Drought and heat	2021–2022	4B	77	AX-94464472	AX-94416930	3.25	5.64	−0.17	76.50	77.50
3	*QSL.iari-4B.2*	Heat	2022–2023	4B	78	AX-94421709	AX-95115092	3.55	6.91	−0.19	77.50	78.50
		Pooled	Pooled	4B	78	AX-94421709	AX-95115092	3.58	6.95	−0.18	77.50	78.50
4	*QSL.iari-5B*	Heat	2021–2022	5B	13	AX-94715923	AX-95166397	3.69	5.76	0.19	12.50	15.50
		Heat	2022–2023	5B	13	AX-94715923	AX-95166397	3.73	7.67	0.19	12.50	15.50
		Heat	2022–2023	5B	13	AX-94715923	AX-95166397	3.94	11.78	0.21	12.50	15.50
		Pooled	Pooled	5B	13	AX-94715923	AX-95166397	3.97	8.07	0.19	12.50	15.50
5	*QSL.iari-6B*	Heat	2021–2022	6B	0	AX-94548199	AX-94998924	4.24	6.13	0.20	0.00	1.50
		Pooled	Pooled	6B	0	AX-94548199	AX-94998924	4.04	7.65	0.19	0.00	1.50
6	*QSL.iari-6D*	Drought and heat	2021–2022	6D	216	AX-95660329	AX-95256931	4.42	7.60	0.20	215.50	216.50
		Heat	2022–23	6D	216	AX-95660329	AX-95256931	4.00	7.84	0.20	215.50	216.50
NDVI												
1	*QNDVI.iari-2A*	Drought and heat	2021–2022	2A	164	AX-94592263	AX-94475771	3.94	8.93	−0.02	159.50	168.50
2	*QNDVI.iari-3A*	Drought and heat	2021–2022	3A	69	AX-94651794	AX-94480950	3.48	10.84	0.01	67.50	72.50
3	*QNDVI.iari-5A*	Heat	2022–2023	5A	344	AX-94445381	AX-95132498	5.83	10.32	−0.01	339.50	347.50
		Heat	Pooled	5A	344	AX-94445381	AX-95132498	4.90	7.51	−0.01	337.50	347.50
4	*QNDVI.iari-5B.1*	Heat	Pooled	5B	7	AX-94544520	AX-94541836	4.43	10.45	0.03	4.50	7.50
5	*QNDVI.iari-5B.2*	Heat	2021–2022	5B	9	AX-94541836	AX-94715923	4.43	7.31	0.02	5.50	12.50
		Drought and heat	2022–2023	5B	10	AX-94541836	AX-94715923	4.34	10.17	0.03	8.50	12.50
		Pooled	Pooled	5B	10	AX-94541836	AX-94715923	5.23	12.33	0.02	8.50	12.50
		Drought and heat	Pooled	5B	11	AX-94541836	AX-94715923	4.15	9.99	0.02	7.50	13.50
		Heat	2022–2023	5B	12	AX-94541836	AX-94715923	3.53	5.37	0.01	7.50	13.50
		Drought and heat	2021–2022	5B	12	AX-94541836	AX-94715923	3.42	6.71	0.01	8.50	13.50
		Drought and heat	Pooled	5B	12	AX-94541836	AX-94715923	4.10	6.87	0.02	7.50	13.50
		Drought and heat	2021–2022	5B	12	AX-94541836	AX-94715923	4.35	10.55	0.01	7.50	13.50
6	*QNDVI.iari-2D.1*	Heat	2021–2022	2D	249	AX-94889714	AX-94671304	4.32	10.16	0.02	239.50	260.50
7	*QNDVI.iari-2D.2*	Drought and heat	2021–2022	2D	277	AX-94458060	AX-94924039	3.67	6.91	0.01	273.50	279.50
8	QNDVI.iari-6D	Drought and heat	Pooled	6D	359	AX-94494277	AX-94532403	3.82	9.87	−0.02	348.50	365.50
Biomass												
1	*QBM.iari-6B*	Heat	2022–2023	6B	196	AX-94932812	AX-94759235	3.62	7.85	0.07	165.50	212.50
		Heat	Pooled	6B	197	AX-94932812	AX-94759235	3.77	9.09	0.07	184.50	209.50
Number of spikelets												
1	*QSN.iari-5B*	Drought and heat	2022–2023	5B	13	AX-94715923	AX-95166397	3.89	9.50	0.64	10.50	13.50

Note: Chr, chromosome on which QTL was identified; Pos, position of the QTL on the linkage map; LOD, logarithm of odd value; Add, additive effect of the QTL; Left CI, start point of confidence interval on the left side of the QTL; Right CI, end point of confidence interval on the right side of the QTL.

### QTL analysis for morphophysiological traits

Four QTLs governing the PH were identified, with LOD scores ranging from 3.36 to 10.46. Under heat and combined stress, three and two QTLs were identified, respectively. The *QPH.iari-6B.1* was identified under both stress conditions. All four QTLs were major, accounting for more than 10% of the phenotypic variance. The *QPH.iari-3B.1* explained the highest Phenotypic Variance Explained (PVE) (19.70%) for plant height. Five QTLs associated with DH were identified, with LOD scores ranging from 4.57 to 12.80. Among these, two QTLs were identified under heat stress and five under combined stress conditions, while *QDH.iari-1B* and *QDH.iari-1D* were identified in both heat and combined stress conditions. Two major QTLs were identified, with *QDH.iari-6D.1* showing the highest PVE (10.57%). Eight QTLs governing the NDVI were identified, with LOD scores ranging from 3.42 to 5.83. Out of this, four QTLs were identified under heat stress and five in combined stress, while *QNDVI.iari-5B.2* was identified in both stress conditions. Notably, five QTLs were major, and among these, *QNDVI.iari-5B.2* exhibited the highest PVE (12.33%).

### QTL mapping for grain yield–related traits

A total of three QTLs were identified for GWPS, with their LOD scores ranging from 3.28 to 4.56. Among these QTLs, two were identified under heat treatment and one under combined stress treatment. Seven QTLs associated with TGW were identified, exhibiting LOD scores ranging from 3.34 to 6.52. Out of the seven, three QTLs were identified under heat and three under combined stress conditions, while one QTL was identified under the pooled mean condition. Among these QTLs, four were major, with *QTGW.iari-7D* demonstrating the highest PVE (13.84%). A total of six QTLs governing the spike length were identified, with LOD scores ranging from 3.25 to 4.24. Out of these, four and three QTLs were identified under heat and combined stress, respectively. The QTL *QSL.iari-5B* accounted for the highest PVE of 11.78%. A single QTL *QBM.iari-6B* linked to BM was identified and accounted for 7.85%–9.09% of the PVE under heat stress. Similarly, one QTL *QSN.iari-5B* governing the number of spikelets was identified under combined stress with 9.50% PVE.

### Stable QTLs and QTL hotspot

In the current study, a total of eight stable QTLs were identified (Table 4). Among these stable QTLs, there was one each for NDVI and TGW, and two each for PH, SL, and DH. Additionally, two distinct QTL hotspots for the various traits were identified (Table 5). One of these hotspots emerged on chromosome 6B within the 0–1 cM position, demonstrating associations with multiple traits, such as PH, TGW, SL, and GWPS. Furthermore, a separate QTL hotspot was identified on chromosome 5B at the position 13 cM, exhibiting specific associations with SL and SN.

**TABLE 4:** List of stable QTLs expressed across the treatments and years.

No.	QTL	Treatment	Season	Chromosome	Position (cM)	Left marker	Right marker	LOD	PVE (%)
1	*QDH.iari-1B*	H, DH	1	1B	129	AX-94897804	AX-94908088	6.35–7.62	5.14–6.37
2	*QDH.iari-1D*	H, DH	1	1D	65	AX-94957467	AX-94517412	11.13–11.69	9.54–9.99
3	*QNDVI.iari-5B.2*	H, DH	1, 2	5B	9	AX-94541836	AX-94715923	3.42–5.23	5.37–12.33
4	*QPH.iari-3B.2*	DH	1, 2	3B	272	AX-94533181	AX-94509316	4.33–6.30	8.69–10.00
5	*QPH.iari-6B.1*	H, DH	1, 2	6B	0	AX-94548199	AX-94998924	3.36–7.75	6.13–11.10
6	*QSL.iari-5B*	H	1, 2	5B	13	AX-94715923	AX-95166397	3.69–3.73	5.76–7.67
7	*QSL.iari-6D*	H, DH	1, 2	6D	216	AX-95660329	AX-95256931	4–4.42	7.6–7.84
8	*QTGW.iari-6B.1*	H, DH	1, 2	6B	0	AX-94548199	AX-94998924	5.01–6.05	10.11–10.43

Note: H, heat stress; DH, combined drought and heat stress.

**TABLE 5:** List of QTL hotspots.

Sl. No.	Chromosome (B)	Position (cM)	Left marker	Right marker	Trait
1	5	13	AX-94715923	AX-95166397	SL, SN
2	6	0–1	AX-94548199	AX-94998924	PH, TGW, SL, GWPS

Note: SL, spike length; SN, number of spikelets; PH, plant height: TGW, thousand-grain weight; GWPS, grain weight per spike.

### Validation of identified QTLs

**FIGURE 7:**
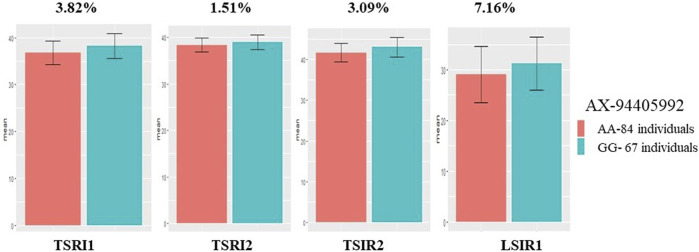
Histogram depicting the average difference between two alleles, AA and GG, associated with the AX-94405992 marker for thousand-grain weight.

Among the 35 QTLs identified, five markers linked to QTLs governing the traits TGW, DH, and NDVI were successfully validated (Table 6). The marker AX-94405992 linked to *QTGW.iari-7D* had AA and GG alleles, wherein group AA had a lower mean TGW (29.03 g) and GG a higher TGW (31.27 g) under heat stress. The mean difference between the two groups was 7.16%, and the marker explains the PVE of 4.07% in validation population. The same marker also showed significant mean differences under TSIR1 (3.09%), TSRI2 (1.51%), and TSRI1 (3.82%) with the PVE of 3.63%, 7.16%, and 7.45%, respectively (Figure 7). The marker AX-94825456 linked to *QTGW.iari-6A.1* had CC and TT alleles, wherein the CC allele group had a higher mean TGW (42.95 g) than the TT allele (41.81 g) under the TSIR1 condition. The mean difference between the two groups was 2.65%, and this marker explains the PVE of 5.50%. The same marker also showed significant mean differences under TSRI1 (3.20%) and TSRI2 (1.59%) with the PVE of 5.95% and 3.97%, respectively. The marker AX-95103934 linked to *QTGW.iari-6B.2* had AA and GG alleles, wherein the AA group had a higher mean TGW (36.84 g) than the GG allele (37.73 g). The mean difference between the two groups was 2.35%, and the marker explains the PVE of 2.77%.

**TABLE 6:** List of successfully validated QTLs in RILs derived from HD3086/HI1500.

Trait	QTL	Treatment*	PVE (%)	Marker	Treatment**	SNP allele	Mean***	*p* value	PVE (%)
TGW	QTGW.iari-7D	LSIR	13.84	AX-94405992	LSIR1	AA	29.03	0.013*	4.07
						GG	31.27		
					TSIR2	AA	41.68	0.0006***	7.6
						GG	43.01		
					TSRI1	AA	36.72	0.0007***	7.45
						GG	38.18		
					TSRI2	AA	38.35	0.0191*	3.63
						GG	38.94		
TGW	*QTGW.iari-6A.1*	Pooled	11.16	AX-94825456	TSIR2	CC	42.95	0.0037**	5.51
						TT	41.81		
					TSRI1	CC	39.81	0.0025**	5.95
						TT	38.54		
					TSRI2	CC	39.01	0.0142*	3.97
						TT	38.39		
TGW	*QTGW.iari-6B.2*	LSRI	5.89	AX-95103934	TSRI1	AA	36.84	0.0448*	2.77
						GG	37.73		
DH	*QDH.iari-1B*	LSIR	5.14	AX-94897804	TSIR1	CC	91.25	0.0481*	2.53
						GG	90.01		
NDVI	*QNDVI.iari-2D.1*	LSIR	10.16	AX-94671304	TSIR2	AA	0.692	0.0426*	2.73
						CC	0.686		

Note: * Treatment in which QTL is identified; ** Treatment in which QTL is validated; *** mean difference between the two allele groups in validation population.

TSIR1, timely sown irrigated condition in 2021–2022.

TSIR2, timely sown irrigated condition in 2022–2023.

TSRI1, timely sown irrigated condition in 2021–2022.

TSRI2, timely sown restricted irrigated condition in 2022–2023.

LSIR, late sown irrigated condition.

LSRI, late sown restricted irrigated condition.

The marker AX-94897804 linked to *QDH.iari-1B* had CC and GG alleles. The CC group had an average 91.25 days to heading, while the GG allele had 90.01 days to heading under the TSRI condition. The difference in DH for the two groups was 1 day, and the PVE of the marker–trait association was a relatively low 2.53%. The marker AX-94671304 linked to *QNDVI.iari-2D.1* had AA and CC alleles, wherein the AA allele group had an average NDVI value of 0.692, and the CC allele group had 0.686. This marker explains a PVE of 2.73%.

### Putative candidate genes in QTL regions

**FIGURE 8:**
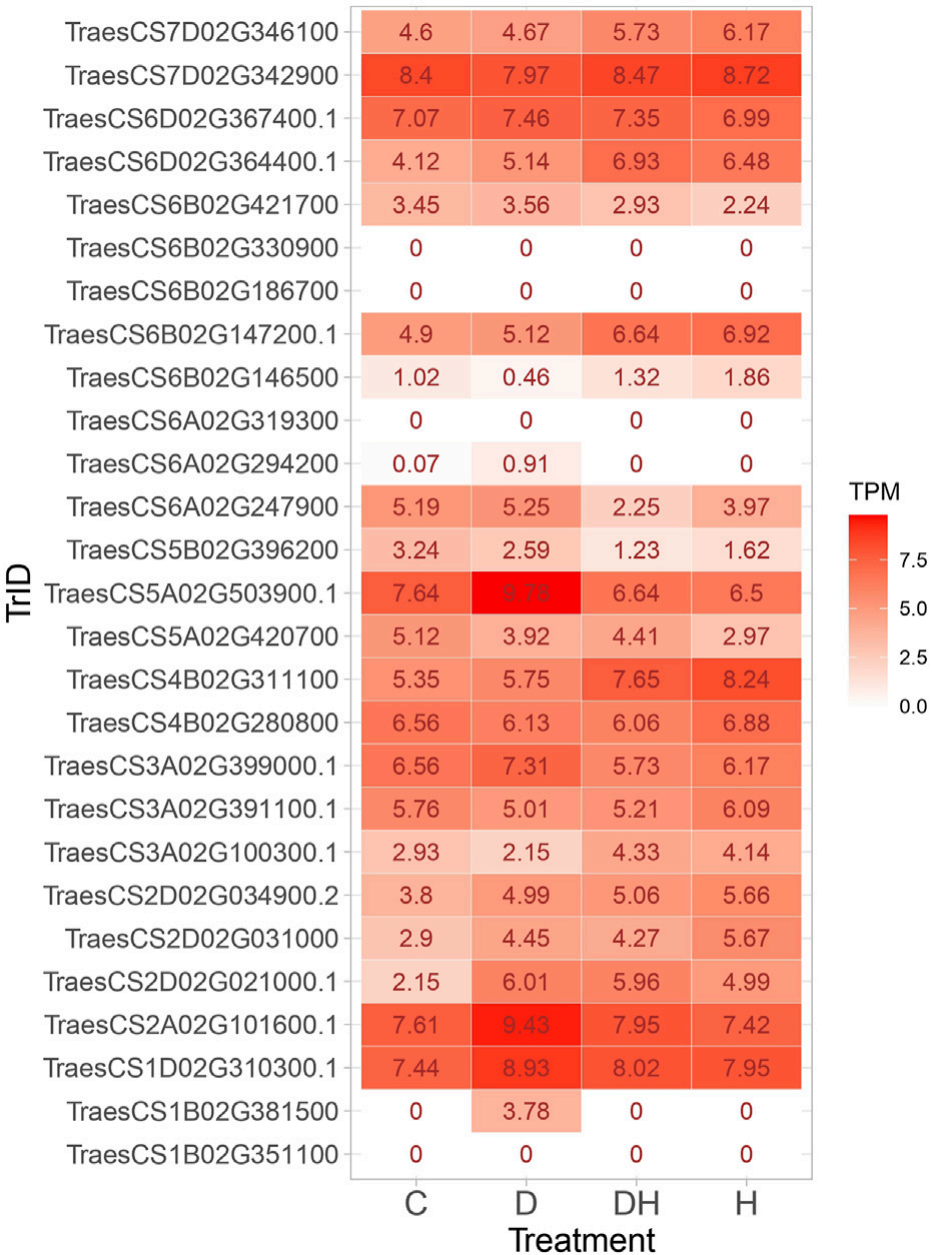
Heatmap showing the expression of transcripts under control, drought, heat, and combined stress conditions.

Candidate genes encoding enzymes that function in antioxidative defense mechanisms by scavenging reactive oxygen species (ROS) were identified (Table 7). Notably, these enzymes encompass peroxidase, trehalose-6-phosphate phosphatase, and S-adenosylmethionine synthase. The investigation revealed transcription factors like MADS-box transcription factor, WRKY DNA-binding protein 58, and ABA-inducible protein that are pivotal for stress signaling and coordinating plants’ adaptive responses to adverse circumstances. Numerous proteins and enzymes engaged in growth processes were identified. These encompass bidirectional sugar transporter (SWEET), flowering locus T3 B1, stress-responsive protein 2, phytochrome, and alpha-amylase, each assuming vital roles in diverse growth-related mechanisms within the plant system. The *in silico* expression analysis identified few transcripts such as TraesCS7D02G342900, TraesCS1D02G310300.1, TraesCS2A02G101600.1, and TraesCS5A02G503900.1 with higher TPM across all conditions. The transcripts TraesCS2D02G021000.1, TraesCS2D02G031000, TraesCS6A02G247900, and TraesCS6B02G421700 displayed higher TPM under stress conditions, implicating their involvement in the stress-responsive pathways (Figure 8).

**TABLE 7:** List of predicted candidate genes and their function in plants.

*QTL*	Transcript ID	Protein	Function	Reference
*QDH.iari-1B*	TraesCS1B02G351100	Flowering locus T3 B1	Controlling the short-day photoperiod response in bread wheat	Zikhal et al. (2017)
	TraesCS1B02G381500	ABA inducible protein	Regulation of responses during abiotic stresses	Singh et al. (2015)
*QDH.iari-1D*	TraesCS1D02G310300.1	Cysteine proteinase inhibitor	Causes premature plant senescence	Seki et al. (2002)
*QTGW.iari-6A.2*	TraesCS6A02G294200	Potassium transporter	Critical for plant drought resistance	Wang et al. (2013)
	TraesCS6A02G319300	Alpha-amylase	Hydrolyzing the endosperm starch into metabolizable sugars	Kaneko et al. (2002)
	TraesCS6A02G247900	S-Adenosylmethionine synthase	Responsible for the production of S-adenosylmethionine, the cofactor essential for various methylation reactions, and production of polyamines and phytohormone ethylene	Sekula et al. (2020)
*QTGW.iari-6B.1*	TraesCS6B02G146500	Peroxidase	Catalyzes various oxidative reactions using hydrogen peroxide	Twala et al. (2020)
	TraesCS6B02G186700	MADS-box transcription factor	Early seed development	Paul et al. (2020)
*QTGW.iari-7D*	TraesCS7D02G342900.1	Homolog of antioxidant 1	Antioxidant against superoxide and hydrogen peroxide	
	TraesCS7D02G346100.1	Pheophytinase	Forward electron transfer, photoprotection, and structural support	Hou (2014)
*QNDVI.iari-5A*	TraesCS5A02G420700	Photosystem II reaction center protein I	Carries out the oxidation (splitting) of water molecules and produces ATP via a proton pump	Lu (2016)
*QNDVI.iari-5B.2*	TraesCS5B02G396200	Phytochrome	Regulates phototropic responses	Kim et al. (2002)
*QNDVI.iari-6D*	TraesCS6D02G364400.1	COBRA-like protein	Cellulose deposition and cell progression in plants by contributing to the microfibril orientation of a cell wall	Sajjad et al. (2023)
	TraesCS6D02G367400.1	Bidirectional sugar transporter SWEET		
*QNDVI.iari-2D.1*	TraesCS2D02G021000.1	Photosystem II D2 protein	Provides the ligands for redox-active cofactors	Stirbet et al. (2019)
	TraesCS2D02G031000.1	Glycosyltransferase	Regulates flavanols accumulation and reactive oxygen species scavenging	Zhao et al. (2019)
	TraesCS2D02G034900.2	Arginase	Mobilizes stored arginine during seed germination and provides nitrogen and carbon sources for the synthesis of other amino acids and polyamines during development and stress management	Splittstoesser (1969)
*QNDVI.iari-3A*	TraesCS3A02G399000.1	Calreticulin-3	Enhances autophagic flux to attenuate cellular stress, likely through alleviation of aberrantly folded proteins	Yang et al. (2019)
	TraesCS3A02G391100.1	Fructose-bisphosphate aldolase	It plays significant roles in biotic and abiotic stress responses and in regulating growth and development processes	Lv et al. (2017)
*QNDVI.iari-2A*	TraesCS2A02G101600.1	Stress-responsive protein 2	Ion scavenging, hypoxia responses, cellular mobility, and regulation of cell growth and development	Chi et al. (2019)
*QBM.iari-6B*	TraesCS6B02G421700	Bidirectional sugar transporter SWEET	Mediates both low-affinity uptake and efflux of sugar across the membrane	Ji et al. (2022)
	TraesCS6B02G330900	Trehalose-6-phosphate phosphatase	Removes the phosphate from trehalose-6-phosphate to produce free trehalose. Trehalose accumulation in plants improves abiotic stress tolerance	Du et al. (2022)
	TraesCS6A02G319300	Alpha-amylase	Hydrolyzing the endosperm starch into metabolizable sugars	Kaneko et al. (2002)
*QSL.iari-4B.2*	TraesCS4B02G311100.1	Lethal leaf spot1	Maintaining cell homeostasis to adapt in various stresses	Tang et al. (2013)
*QSL.iari-4B.1*	TraesCS4B02G280800.1	40S ribosomal protein S27	Synthesis of proteins in the cell	Anger et al. (2013)
*QSL.iari-5B*	TraesCS5B02G401800.1	WRKY DNA-binding protein 58	Plant-specific transcription factor group, playing important roles in many different response pathways during drought, salinity, alkalinity, and heat stress	Li et al. (2020)

## Discussion

Abiotic stresses such as drought, heat, and their combination pose significant challenges to crop productivity and food security worldwide. The study findings demonstrated a notable decline in plant height, biomass, and yield when exposed to heat and combined stress conditions. The most significant decrease was observed under the combined stress condition, in accordance with previous studies by Asseng et al. (2015), Akter and Rafiqul Islam (2017), and Qaseem et al. (2019). This reduction in plant height could be attributed to changes in hormonal regulation and hindered cell elongation, commonly observed under environmental stress conditions (Omar et al., 2023). Interestingly, altered days to heading under late sown conditions were observed. This adaptive response is consistent with the phenomenon of heat-induced early flowering in plants to avoid stress during sensitive growth stages (Poudel and Poudel, 2020). Additionally, reductions in spike length and the number of spikelets were observed under all stress conditions, highlighting the inhibitory effect of heat and combined stress on spikelet development and elongation. Similar responses were also reported by Djanaguiraman et al., 2020 and Shenoda et al., 2021. Under the late sown condition, the anthesis and grain-filling stage coincided with higher temperature stress, hence a decrease in the number of spikelets and spike length was observed. The decrease in thousand-grain weight and grain weight per spike under stress further contributed to reduced overall yield, showing the adverse effect of abiotic stresses on grain development and filling (Mirbahar et al., 2009; Devate et al., 2022). Understanding the molecular and physiological mechanisms regulating spike and seed development under stress is crucial for improving wheat yield under challenging environmental conditions.

The correlation analysis provides valuable insights into the associations between component traits with grain yield. Traits such as biomass, thousand-grain weight, and grains per spike demonstrate significant positive correlations with yield, indicating that plants with higher biomass, larger grain size, and more grains per spike tend to have increased grain yield (Baillot et al., 2018; Philipp et al., 2018). Conversely, days to heading exhibited significant negative associations with yield, suggesting that late heading negatively impacts grain yield (Ullah et al., 2021). In the late heading genotypes, the reproductive phase is accelerated under stress conditions leading to poor grain filling, hence reducing the yield potential (Ludwig and Asseng, 2010; Shavrukov et al., 2017). These findings can aid in the selection of component traits of yield under different environmental conditions, particularly under stress.

The genetic linkage map spanned a length of 6,769.45 cM with the marker density ranging from 2.28 cM/marker in 1A to 14.21 cM/marker in 5D, with an average marker density of 5.52 cM/marker (Puttamadanayaka et al., 2020; Khaled et al., 2022; Manjunath et al., 2023). The variation in marker density across chromosomes reflects differences in recombination rates and genetic distances between the markers (Zhang et al., 2019). Genome B displays the highest number of markers, followed by genomes A and D (Yang et al., 2017; Gutierrez-Gonzalez et al., 2019; Manjunath et al., 2023). This trend can be attributed to the greater diversity in A and B genomes than in the D genome. Genetic differences between parental genomes could also impact marker distribution. Importantly, the prevalence of B genome markers might be due to the 35 k chip used, which includes a larger representation of B genome markers.

A total of 35 QTLs were identified that included various traits such as NDVI (eight QTLs), TGW (seven QTLs), SL (six QTLs), DH (five QTLs), PH (four QTLs), GWPS (three QTLs), BM (one QTL), and SN (one QTL). Among these, nine QTLs were specific to heat stress, 13 were specific to combined stress conditions, and six QTLs were consistently present across both stresses. Out of the five QTLs for DH, the *QDH.iari-6D.1* showed a high PVE of 10.57%, making it a potential target for future breeding efforts. The QTLs for DH were located on chromosomes 1B, 6B, 1D, and 6D. Previous works have reported QTLs on chromosomes 1B (Tahmasebi et al., 2016), 6B (Lopes et al., 2012), 1D (Tahmasebi et al., 2016; Pankaj et al., 2021; Devate et al., 2022), and 6D (Puttamadanayaka et al., 2020; Devate et al., 2022). Among the five QTLs governing DH, there were two stable ones. The *QDH.iari-1B* harbors the candidate gene that codes for protein flowering locus T3 B1 (TaFT3-B1), which is responsible for regulating the short-day photoperiod response in bread wheat. A higher copy number of TaFT3-B1 was linked to early flowering (Zikhali et al., 2017). Among the four major QTLs identified for PH, three were stable. These QTLs were present on chromosomes 3A, 3B, and 6B. The QTLs for PH were also reported on chromosomes 3A (Lopes et al., 2012; Khaled et al., 2022), 3B (Khaled et al., 2022), and 6B (Lopes et al., 2012; Devate et al., 2022; Khaled et al., 2022) in similar previous works. Among the eight QTLs mapped for NDVI, the major and stable QTL *QNDVI.iari-5B.2* with 12.33% PVE could be potential targets for further breeding efforts. The genomic region of QTL *QNDVI.iari-5B.2* encodes phytochrome that regulates phototropic responses (Kim et al., 2002). QTLs have also been reported in previous works on chromosomes 2A (Puttamadanayaka et al., 2020; Devate et al., 2022), 5A (Puttamadanayaka et al., 2020), 5B (Devate et al., 2022), and 2D (Puttamadanayaka et al., 2020; Devate et al., 2022).

This study identified seven QTLs for TGW, among which *QTGW.iari-7D* is a major QTL with the highest PVE of 13.84%. QTLs for TGW were also identified on chromosomes 6A, 6B, 7B, 6D, and 7D. Previous studies have reported QTLs on chromosomes 6A (Goel et al., 2019; Negisho et al., 2022) and 7A (Goel et al., 2019; Negisho et al., 2022), while 7B, 6D, and 6D seem to be novel. The stable QTL *QTGW.iari-6A.2* codes for enzymes that include S-adenosylmethionine synthase, alpha-amylase, and potassium transporter. S-Adenosylmethionine synthase was found to be responsible for the synthesis of polyamines, which act as priming agents and stimulate antioxidant defense (Chen et al., 2019). Among the three QTLs identified for GWPS, the *QGWPS.iari-7D* was found to be the most influential, explaining a significant phenotypic variance of 8.67%. Additionally, QTLs were also identified on chromosomes 6B and 7B for GWPS. Previous works have reported QTLs on chromosomes 6B (Schmidt et al., 2020) and 7B (Schmidt et al., 2020; Devate et al., 2022). A total of six QTLs were identified, out of which, three QTLs were major, among which *QSL.iari-5B* showed the highest PVE of 11.78%. The QTL on chromosome 5A was also reported by Zhou et al., 2017, while QTLs on chromosomes 4B, 5B, 6B, and 6D might be novel. A single QTL *QBM.iari-6B* was identified for BM, and genomic regions of this QTL contain genes encoding bidirectional sugar transporter SWEET, trehalose-6-phosphate phosphatase, and alpha-amylase enzyme. The bidirectional sugar transporter SWEET mediates the low-affinity uptake and efflux of sugar across the membrane (Ji et al., 2022). The enzyme trehalose-6-phosphate phosphatase removes the phosphate from trehalose-6-phosphate, leading to the production of free trehalose which enhances abiotic stress tolerance in plants (Du et al., 2022).

The validation of QTLs identified in one population (GW322/KAUZ) using another set of diverse population (HD3086/HI1500) is a crucial step in confirming the robustness and applicability of these markers for traits under study (Singroha et al., 2021). Five QTLs linked to traits TGW, DH, and NDVI were successfully validated, indicating their practical utility in plant breeding through marker-assisted selection. Marker AX-94405992 linked to the QTL *QTGW.iari-7D* showed significant marker trait linkages under control, drought, and heat stress, suggesting its potential for improving grain weight across various environments. The mean difference for the two allele groups of this ranged from 1.51% to 7.16% (Ji et al., 2022). Furthermore, marker AX-94825456 linked to the QTL *QTGW.iari-6A.1* governing TGW was successfully validated under control and drought treatments, indicating its impact on grain weight. The mean difference between the two alleles of the marker groups ranged from 1.58% to 3.19%. The marker AX-95103934 linked to the QTL *QTGW.iari-6B.2* exhibited a significant marker trait linkage with TGW, and the mean difference between the two allele groups was 2.35%. Similar validation for TGW on chromosomes 1B, 4B, and 7A was done by Cao et al. (2020). The marker AX-94897804 linked to QTL *QDH.iari-1B* can be used in breeding programs aimed at developing varieties with optimized flowering time under stress conditions, which is a key factor in determining wheat adaptation and performance under changing environmental conditions. Additionally, marker AX-94671304 linked to trait NDVI was also successfully validated and can be used for improving the stay-green trait (Taria et al., 2023).

## Conclusion

The knowledge of QTLs associated with component traits of yield under drought and heat stress is of prime importance for developing climate-resilient varieties. The current investigation revealed that most of the traits exhibited a normal distribution except NDVI at maturity stage, which displayed a skewed distribution due to the stay-green expression. Combined stress significantly reduced the yield by affecting the component traits that contribute to the overall yield. Furthermore, the traits BM, TGW, and GWPS showed a significant positive correlation with PY, while DH and SN displayed a negative correlation with PY, underscoring the significance of these traits in relation to yield under stress conditions. Out of the 35 identified QTLs for various traits, nine were specific to heat stress, 13 to combined stress, and six were found in both heat and combined stress conditions. Furthermore, eight stable QTLs and two QTL hotspots each containing QTLs for multiple traits on chromosomes 5B and 6D were identified. The genomic regions of QTLs harbor candidate genes encoding antioxidant enzymes, transcription factors, and nutrient transporters, which play a role in abiotic stress tolerance. Five QTLs were successfully validated, encompassing three for TGW, one for NDVI, and one for PH, making them viable candidates for marker-assisted selection in the development of climate-resilient varieties.

## Data Availability

The original contributions presented in the study are included in the article/Supplementary material, further inquiries can be directed to the corresponding authors.
